# Supplementary material for: Complexome profiling of the Chlamydomonas psb28 mutant reveals TEF5 as an early PSII assembly factor
Source: Plant Cell. 2025 Mar 17;37(6):koaf055. doi: 10.1093/plcell/koaf055 (PMC12164590; doi:10.1093/plcell/koaf055)
Supplement: koaf055_Supplementary_Data [file koaf055_supplementary_data.zip › psb28_interactive.zip!/plots/intensity/qualityPlots/peptides/TSP4_Cre10.g420550.t1.1.html]

### Cre10.g420550.t1.1

Trivial name: TSP4  
SeqCoverage [median]: 5.40  
Mass: 52.51 kDa  
  
 

| Sequence | MissedCleavage | Mass [Da] | wtIntensity | muIntensity | Score |
| --- | --- | --- | --- | --- | --- |
| AGVRPGDVIAAVDGK | 1 | 1423.78 | 0.00 | 1492.16 | 30.54 |
| VVAPTEGGPSER | 0 | 1197.60 | 0.00 | 1588.23 | 71.35 |
|  |  |  | 0.0 | 3080.4 |  |
